# Editorial: Advanced Strategies to Reduce the Nitrate Content in Vegetables

**DOI:** 10.3389/fpls.2021.765636

**Published:** 2021-09-28

**Authors:** Angelo Signore, Luke Bell, Pietro Santamaria, Marie-Christine Van Labeke

**Affiliations:** ^1^Department of Agricultural and Environmental Science, University of Bari Aldo Moro, Bari, Italy; ^2^School of Agriculture, Policy and Development, University of Reading, Reading, United Kingdom; ^3^Department Plants and Crops, Faculty of Bioscience Engineering, Ghent University, Ghent, Belgium

**Keywords:** nitrates, light emitting diodes (LED), vegetable, proton pumps, floating system, ebb and flow system, nitrate reductase, *Mesembryanthemum crystallinum*

Nitrogen is an essential element for plant life, as it is a component of amino acids, proteins, nucleic acids (DNA and RNA), and chlorophyll. Nitrogen is not only important for plants; it is the most abundant element in the atmosphere, and its use by humans in industry has transformed our species, our food production systems, and the planet.

The uptake of nitrogen by crops is done in two ion forms, namely as ammonium (${\text{NH}}_{4}^{+}$) and nitrate (${\text{NO}}_{3}^{-}$). The nitrate is readily absorbed by plants, but at the same time it can be lost just as easily into the soil by leaching, since it dissolves in water and leaches out of the soil. This has potentially damaging effects, polluting underground waterways, rivers, and streams.

At crop level, nitrogen is used for nutrition, but it is also an important factor on a qualitative level, being associated with the coloration of plant leaves. For this reason, farmers tend to over-fertilize the crops, particularly leafy vegetables, as a pale green color depreciates the product commercially. However, such a practice may create problems of high nitrate concentration in fresh vegetables, with potential health implications for consumers, since nitrate ingested can be converted into nitrite and nitrosamines in the stomach, which is hypothesized to be harmful for human health.

For this reason, the European Union amended the Commission Regulation (EU) No 1258/2011 of 2 December 2011, amending Regulation (EC) No 1881/2006, concerning maximum levels for nitrates in foodstuffs, that includes several leafy vegetables. However, it is not always easy to maintain the nitrate concentration below the legal limits, particularly during periods of low light intensity and temperatures during the autumn-winter cycle; especially in greenhouse productions and at northern latitudes (e.g., in the United Kingdom and Scandinavia). Hence, an integrated strategy is necessary to avoid exceeding these limits that would make the foodstuff not legally marketable.

The Research Topic “*Advanced Strategies to Reduce the Nitrate Content in Vegetables*” had the objective to explore different approaches with the aim to reduce the nitrate content in vegetables, by considering the several aspects that may have a role in reducing nitrate content in vegetables.

Liang and Zhang realized a mini review focusing on the physiological aspects of nitrate uptake by describing the mechanisms underlying the transport and distribution of ${\text{NO}}_{3}^{-}$ in plants, with the purpose to furnish suggestions to breeders for the production of leafy vegetables with lower ${\text{NO}}_{3}^{-}$ content. This is an important aspect, from both an environmental and a food safety point of view.

On another topic, Conversa et al. took into consideration crop management practices in a soilless cultivation system. Two levels of electroconductivity (EC), two periods of cultivation and two soilless systems on lettuce and endive were compared. These crops are important and used as baby leaf products in the ready-to-eat (RTE) sector. The authors reported that the degree of nitrate concentration reduction after N removal from nutrient solution, without observing a decrease in yield, varied in function of several parameters, and suggested that a more precise tuning is required to adjust the duration of nitrogen removal to the soilless systems used.

He and Qin reported how a reduction of nitrate supply, on *Mesembryanthemum crystallinum*—a plant that is gaining popularity for human consumption, may affect some parameters such as nitrogen metabolism, the efficient use of light for photosynthesis and the nutritional value of the crop. They highlighted that ${\text{NO}}_{3}^{-}$ withdrawal from the nutrient solution before harvesting may be a good strategy to reduce NO${}_{3}^{-}$ concentration in the shoot.

Finally, Signore et al. focused their research on rocket crops, which are important species for the RTE sector, and act as “hyper accumulators” for nitrate. The authors tested the hypothesis that light emitting diode (LED) spectra may have an influence in reducing concentration of ${\text{NO}}_{3}^{-}$ in leaves and improve nutritional traits by increasing glucosinolate content. Their results showed that red light is effective in reducing nitrate concentration by increasing nitrate reductase activity, even if the extent of reduction is species dependent. The content of glucosinolates was significantly determined by the species, with *Diplotaxis tenuifolia* showing higher concentrations relative to *Eruca sativa*.

## Author Contributions

All authors listed have made a substantial, direct and intellectual contribution to the work, and approved it for publication.

## Conflict of Interest

The authors declare that the research was conducted in the absence of any commercial or financial relationships that could be construed as a potential conflict of interest.

## Publisher's Note

All claims expressed in this article are solely those of the authors and do not necessarily represent those of their affiliated organizations, or those of the publisher, the editors and the reviewers. Any product that may be evaluated in this article, or claim that may be made by its manufacturer, is not guaranteed or endorsed by the publisher.

